# Factors associated with the development, severity, and resolution of post COVID-19 condition in adults living in Canada, January 2020 to August 2022

**DOI:** 10.17269/s41997-024-00958-7

**Published:** 2024-11-01

**Authors:** Dianne Zakaria, Alain Demers, Nicholas Cheta, Samina Aziz, Peri Abdullah

**Affiliations:** Centre for Surveillance and Applied Research, Health Promotion and Chronic Disease Prevention Branch, Public Health Agency of Canada, Ottawa, ON Canada

**Keywords:** SARS-CoV-2, Post COVID-19 condition, Long COVID, Symptoms, Incidence, Prevalence, Severity, Resolution, Multivariable modeling, Canada, Cross-sectional survey, SARS-CoV-2, Syndrome post-COVID-19, COVID longue, Symptômes, Incidence, Prévalence, Gravité, Résolution, Modélisation multivariée, Canada, Enquête transversale

## Abstract

**Objectives:**

We aimed to characterize the burden of post COVID-19 condition (PCC) among adults in Canada and identify factors associated with its occurrence, severity, and resolution.

**Methods:**

We used self-report data from a population-based cross-sectional probability survey of adults in Canada conducted between April and August 2022. Incidence and prevalence of PCC were estimated using confirmed infections, as well as confirmed and suspected combined. Multivariable modeling using confirmed cases identified associated factors.

**Results:**

As of August 2022, 17.2% (95% CI 15.7, 18.8) of adults with confirmed infections and 16.7% (95% CI 15.5, 18.0) of adults with confirmed or suspected infections experienced PCC, translating to 3.3% (95% CI 3.0, 3.6) and 4.4% (95% CI 4.1, 4.8) of all adults, respectively. Age less than 65 years (aORs of 1.75 to 2.14), more pre-existing comorbidities (aORs of 1.75 to 3.57), and a more severe initial infection (aORs of 3.52 to 9.69) were all associated with higher odds of PCC, while male sex at birth (aOR = 0.54, 95% CI 0.41, 0.70), identifying as Black (aOR = 0.23, 95% CI 0.11, 0.51), and being infected after 2020 (aORs of 0.24 to 0.55) were associated with lower odds. Those residing in a rural area (aOR = 2.31, 95% CI 1.35, 3.93), or reporting a disability (aOR = 2.87, 95% CI 1.14, 7.25), pre-existing chronic lung condition (aOR = 5.47, 95% CI 1.85, 16.12) or back problem (aOR = 2.34, 95% CI 1.26, 4.36), or PCC headache (aOR = 2.47, 95% CI 1.60, 3.83) or weakness (aOR = 2.27, 95% CI 1.41, 3.68) had higher odds of greater limitations in daily activities, while males had lower odds (aOR = 0.54, 95% CI 0.34, 0.85). Two or more pre-existing chronic conditions (aHRs from 0.33 to 0.38), or PCC symptoms relating to the heart (aHR = 0.25, 95% CI 0.07, 0.90), brain fog (aHR = 0.44, 95% CI 0.23, 0.86), or stress/anxiety (aHR = 0.48, 95% CI 0.24, 0.96) were associated with a decreased rate of symptom resolution.

**Conclusion:**

Over the first two and a half years of the pandemic, a substantial proportion of infected adults in Canada reported PCC. Females and people with comorbidities were disproportionately impacted.

**Supplementary Information:**

The online version contains supplementary material available at 10.17269/s41997-024-00958-7.

## Résumé

**Objectifs:**

Nous avons cherché à caractériser le fardeau du syndrome post-COVID-19 (SPC) chez les adultes au Canada et à identifier les facteurs associés à son apparition, sa gravité et sa résolution.

**Méthodes:**

Nous avons utilisé les données d’auto-évaluation d’une enquête probabiliste transversale basée sur la population d’adultes au Canada menée entre avril et août 2022. L’incidence et la prévalence du SPC ont été estimées à l’aide d’infections confirmées, ainsi que d’infections confirmées et soupçonnées combinées. La modélisation multivariée à l’aide des cas confirmés a permis d’identifier des facteurs leur étant associés.

**Résultats:**

En août 2022, 17,2% (IC à 95%: 15,7, 18,8) des adultes atteints d’infections confirmées et 16,7% (IC à 95%: 15,5, 18,0) des adultes atteints d’infections confirmées ou soupçonnées ont développé un SPC, soit 3,3% (IC à 95%: 3,0, 3,6) et 4,4% (IC à 95%: 4,1, 4,8) de tous les adultes, respectivement. Être âgé de moins de 65 ans (aRC de 1,75 à 2,14), un plus grand nombre de comorbidités préexistantes (aRC de 1,75 à 3,57) et une infection initiale plus grave (aRC de 3,52 à 9,69) étaient tous associés à une probabilité plus élevée de SPC, alors qu’être un garçon à la naissance (aRC = 0,54, IC à 95%: 0,41, 0,70), s’identifier comme Noir (aRC = 0,23, IC à 95%: 0,11, 0,51) et le fait d’être infecté après 2020 (aRC de 0,24 à 0,55) étaient associés à des probabilités plus faibles. Les personnes résidant dans une région rurale (aRC = 2,31, IC à 95%: 1,35 à 3,93) ou déclarant une incapacité (aRC = 2,87, IC à 95%: 1,14 à 7,25), une affection pulmonaire chronique préexistante (aRC = 5,47, IC à 95%: 1,85 à 16,12), des problèmes de dos (aRC = 2,34, IC à 95%: 1,26 à 4,36), des maux de tête liés au SPC (aRC = 2,47, IC à 95%: 1,60 à 3,83) ou une faiblesse liée au SPC (aRC = 2,27, IC à 95%: 1,41, 3,68) avaient une probabilité plus élevée de limitations plus importantes des activités quotidiennes, tandis que les hommes avaient une probabilité plus faible (aRC = 0,54, IC à 95%: 0,34, 0,85). Le fait d’avoir deux maladies chroniques préexistantes ou plus (aRR de 0,33 à 0,38) ou des symptômes de SPC liés au cœur (aRR = 0,25, IC à 95%: 0,07, 0,90), au brouillard cérébral (aRR = 0,44, IC à 95%: 0,23, 0,86) ou au stress/anxiété (aRR = 0,48, IC à 95%: 0,24, 0,96) étaient associés à une diminution du taux de résolution des symptômes.

**Conclusion:**

Au cours des deux premières années et demie de la pandémie, une proportion importante d’adultes infectés au Canada a déclaré un SPC. Les femmes et les personnes avec des comorbidités ont été touchées de manière disproportionnée.

## Introduction

Although most people infected with SARS-CoV-2 uneventfully recover, a significant proportion may experience symptoms beyond the acute infection stage. These longer-term, wide-ranging symptoms are commonly referred to as post COVID-19 condition (PCC) or long COVID with durations of 4 or 12 weeks commonly required to satisfy case definitions (CDC, 2023; NICE, SIGN and RCGP, 2022; Soriano et al., 2022). In this study, we use PCC to refer to symptoms present three or more months after SARS-CoV-2 infection. We use “3 months” instead of “12 weeks” for consistency with the data source used in our study. Two recent systematic reviews estimated the pooled prevalence of PCC after confirmed or suspected infection during the pre-Omicron period at 25.6–26.9% in primarily non-hospitalized or community-dwelling populations (O’Mahoney et al., 2023; Woodrow et al., 2023). Female sex, older age, infection with pre-Omicron variants, more severe acute infection, high body mass index, comorbidities, smoking, and incomplete vaccination prior to infection have been associated with an increased risk of developing PCC and a slower rate of recovery (Hedberg & Nauclér, 2024; Jennings et al., 2023; Notarte et al., 2022; Perlis et al., 2023; Pillay et al., 2022; Robineau et al., 2022; Tsampasian et al., 2023). In addition, brain fog, shortness of breath, and a greater number of acute symptoms have also been associated with a slower resolution (Perlis et al., 2023; Robineau et al., 2022).

Considering the average infection-acquired SARS-CoV-2 seroprevalence in Canada’s ten provinces was 81.8% as of the end of October 2023 (Swail et al., 2023), the potential burden of PCC on afflicted individuals and their families, the healthcare system, and the economy, through lost productivity, could be substantial. We aimed to characterize the burden of PCC in adults living in Canada and identify factors associated with its development, severity, and resolution. Our findings will contribute to an evidence base informing public health guidance, prevention, treatment and management strategies, resource allocation, and future research. Identifying factors associated with the development, severity, and recovery from PCC allows for targeted earlier intervention with the goal of minimizing longer-term impacts.

## Methods

### Data source

**Fig. 1:**
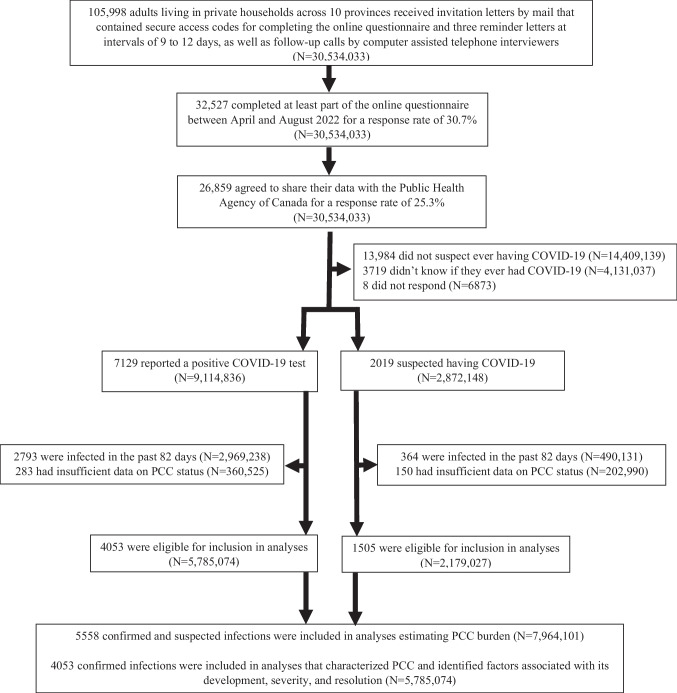
Flow of adults from recruitment to inclusion in analyses. The data source is the Canadian COVID-19 Antibody and Health Survey – Cycle 2. Weighted numbers may not sum to total due to rounding. *COVID-19* coronavirus disease 2019, *N* weighted number of adults, *PCC* post COVID-19 condition

We used the second cycle of the Canadian COVID-19 Antibody and Health Survey (CCAHS-2), a large population-based cross-sectional probability survey conducted between April and August 2022 by means of an online questionnaire (Statistics Canada, 2023). Individuals younger than 18 years of age, persons living in the three territories or on reserves and other Indigenous settlements in the provinces, members of the Canadian Forces living on a base, institutionalized people, and residents of certain remote regions were excluded. In total, 105,998 adults were invited to participate, 32,527 (30.7%) responded, and 26,859 (25.3%) agreed to share their data with the Public Health Agency of Canada (Fig. 1).

### Outcomes of interest

We defined PCC as persistent, recurrent, or new symptoms present three or more months after first testing positive for (e.g., polymerase chain reaction or rapid antigen test) or suspecting a SARS-CoV-2 infection. This included symptoms from the initial infection that lasted three or more months or symptoms that developed after an initial recovery. For adults testing positive, we did not have information on the timing of the test relative to the occurrence of acute symptoms, so we consistently used the date of testing as an estimate of the date of infection. Since 3 months of follow-up was necessary to ascertain PCC, our analytical sample was limited to respondents who indicated being infected more than 82 days prior to questionnaire completion and did not provide a conflicting infection date (unweighted *n* = 5558, Fig. 1). We used 82 rather than 89 days to account for the imprecision of the collected infection date which was self-reported as occurring in the early (assigned 5th day of the month), middle (15^th^), or late (25^th^) part of the month.

PCC burden was measured using two approaches to provide a range of plausible values. The first included confirmed infections, while the second included confirmed and suspected infections to acknowledge limitations in testing capacity early in the pandemic. Cumulative incidence was defined as the proportion of infected adults ever experiencing PCC. Complete and point prevalence were defined as the proportion of adults (infected and not infected) ever experiencing PCC and continuing to experience PCC at the time of questionnaire completion, respectively. When estimating prevalence, adults infected less than 3 months prior to completing their questionnaire were classified as not having PCC.

In addition to the development of PCC, we also examined its severity and time to resolution. Severity of PCC was quantified using self-reported limitations in daily activities due to PCC rated on a 5-point ordinal scale and trichotomized for analysis (never/rarely, sometimes, often/always). Time to resolution was quantified using two variables. The first captured duration of symptoms at time of questionnaire completion as categorized uneven intervals: < 2 months, 2 to < 3 months, 3 to < 6 months, 6 months to < 1 year, and ≥ 1 year. The second indicated whether the respondent continued to experience PCC at the time of questionnaire completion, resulting in a right censored measure of duration. The midpoints of the duration categories were used to estimate duration and all respondents selecting 1 year or longer were censored at 365 days due to the open upper limit of the interval.

### Independent variables

Examined variables captured the following: sociodemographics, health status, vaccination status, and infection-related information (Tables 1, 2, 3). Health status variables included presence of each of 21 pre-existing chronic conditions and 34 pre-existing chronic symptoms, as well as the total number of each, and body mass index (Table 2). Pre-existing chronic conditions were defined as conditions lasting or expected to last at least 6 months that were diagnosed by a healthcare professional up to and including the month of SARS-CoV-2 infection. Number of pre-existing chronic conditions as of the month of infection excludes cancer because the date of cancer diagnosis is unknown. Because of the non-specific nature of PCC symptoms and the overlap in the types of chronic and PCC symptoms examined in the questionnaire, we were concerned that some of the chronic symptoms reported by respondents could be PCC symptoms. To minimize this misclassification, we defined pre-existing chronic symptoms as symptoms lasting or expected to last at least 6 months that first started at least 2 months prior to the month of infection. Methods used to impute dates of diagnosis and onset of chronic conditions and symptoms, respectively, and dates of COVID-19 vaccinations are detailed in the supplementary methods.

**Table 1:** Sociodemographic characteristics of adults self-reporting a positive COVID-19 test three or more months prior to questionnaire completion by post COVID-19 condition status, Canada, January 2020 to August 2022

Characteristics	All *n* = 4053		No PCC *n* = 3328		PCC *n* = 725		*p*-value
	Estimate	95% CI	Estimate	95% CI	Estimate	95% CI	
Sex at birth							< 0.0001
Male	49.9	(48.1, 51.8)	52.8	(50.7, 54.9)	36.1	(31.3, 41.0)	
Female	50.1	(48.2, 51.9)	47.2	(45.1, 49.3)	63.9	(59.0, 68.7)	
Gender							< 0.0001
Man	49.8	(48.0, 51.7)	52.7	(50.6, 54.7)	36.2	(31.4, 41.2)	
Woman	49.9	(48.1, 51.8)	47.2	(45.1, 49.3)	63.1	(58.0, 67.9)	
Other	-^a^	-	-	-	-	-	
Age at infection, years (mean)	41.4	(40.8, 41.9)	41.5	(40.9, 42.0)	40.9	(39.6, 42.2)	0.4468
Age at infection (years)							0.4068
15‒34	39.3	(37.4, 41.1)	39.0	(37.0, 41.1)	40.4	(35.7, 45.3)	
35‒49	32.1	(30.5, 33.8)	32.5	(30.6, 34.4)	30.5	(26.6, 34.5)	
50‒64	21.0	(19.6, 22.5)	20.7	(19.1, 22.3)	22.8	(19.2, 26.6)	
65 +	7.6	(6.8, 8.4)	7.8	(6.9, 8.8)	6.4	(4.7, 8.4)	
Pregnant	1.0	(0.6, 1.5)	1.1	(0.7, 1.7)	-	-	-
Sexual orientation							0.0110
Heterosexual	92.9	(91.7, 94.1)	93.5	(92.1, 94.7)	90.2	(86.6, 93.1)	
Lesbian, gay, bisexual, other	5.4	(4.5, 6.5)	4.8	(3.8, 6.0)	8.6	(5.8, 12.1)	
Unknown	1.6	(1.1, 2.3)	1.7	(1.1, 2.5)	-	-	
Highest education completed in household							0.2977
Less than high school	2.8	(2.2, 3.5)	2.9	(2.2, 3.7)	2.4	(1.4, 3.9)	
High school diploma	10.4	(9.2, 11.7)	9.9	(8.6, 11.2)	12.7	(9.6, 16.4)	
Trade certificate or diploma	7.3	(6.4, 8.2)	7.3	(6.4, 8.4)	6.9	(4.7, 9.8)	
College or university certificate, diploma, or degree	79.6	(77.9, 81.1)	79.9	(78.2, 81.5)	78.0	(73.6, 81.9)	
Ethnicity							< 0.0001
First Nations only	1.8	(1.3, 2.4)	1.4	(0.9, 2.0)	3.9	(2.3, 6.1)	
Metis only	1.4	(1.0, 1.8)	1.4	(1.0, 1.9)	-	-	
White only	68.1	(65.9, 70.2)	67.7	(65.2, 70.1)	70.0	(65.1, 74.6)	
Black only	3.5	(2.7, 4.5)	4.0	(3.1, 5.1)	1.2	(0.6, 2.3)	
Arab or West Asian only	3.8	(3.0, 4.7)	4.1	(3.2, 5.1)	-	-	
South Asian only	7.4	(6.1, 8.8)	7.0	(5.7, 8.6)	9.0	(5.8, 13.2)	
East or Southeast Asian only	9.0	(7.6, 10.5)	9.6	(8.1, 11.2)	6.2	(3.8, 9.4)	
Latin American only	2.6	(1.9, 3.4)	2.7	(2.0, 3.7)	-	-	
Other racial or cultural group^b^	2.5	(1.9, 3.2)	2.1	(1.4, 2.8)	4.5	(2.6, 7.2)	
Dwelling type							0.0247
Single detached, double, or duplex	71.5	(69.5, 73.5)	71.2	(69.0, 73.4)	73.0	(68.1, 77.5)	
Row, terrace, or low-rise apartment	19.6	(18.0, 21.3)	20.4	(18.6, 22.3)	15.4	(12.1, 19.2)	
High-rise apartment	7.5	(6.2, 8.9)	6.9	(5.6, 8.4)	10.4	(7.0, 14.5)	
Other	1.4	(1.0, 2.0)	1.5	(1.0, 2.1)	-	-	
Urban/rural residence							0.5577
Urban	86.3	(85.1, 87.4)	86.2	(84.8, 87.4)	87.1	(84.0, 89.8)	
Rural	13.7	(12.6, 14.9)	13.8	(12.6, 15.2)	12.9	(10.2, 16.0)	
Remoteness of residence							0.6352
Easily accessible	79.5	(78.2, 80.8)	79.4	(78.0, 80.7)	80.2	(76.9, 83.2)	
Accessible	16.1	(15.0, 17.3)	16.4	(15.1, 17.7)	14.7	(12.1, 17.6)	
Less accessible to very remote	3.9	(3.4, 4.6)	3.8	(3.2, 4.6)	4.4	(3.0, 6.4)	
Unknown	0.4	(0.3, 0.7)	0.4	(0.2, 0.7)	-	-	
Province of residence							0.0034
Newfoundland and Labrador	1.3	(0.9, 1.7)	1.2	(0.9, 1.7)	1.4	(0.7, 2.5)	
Prince Edward Island	0.3	(0.2, 0.6)	0.3	(0.1, 0.5)	0.5	(0.1, 1.3)	
Nova Scotia	2.4	(1.9, 2.9)	2.3	(1.9, 2.9)	2.6	(1.6, 4.1)	
New Brunswick	2.0	(1.6, 2.5)	2.0	(1.6, 2.6)	2.0	(1.1, 3.4)	
Quebec	26.1	(24.6, 27.7)	27.0	(25.4, 28.7)	22.0	(18.4, 25.9)	
Ontario	35.3	(33.5, 37.2)	35.6	(33.4, 37.7)	34.2	(29.0, 39.7)	
Manitoba	3.4	(2.9, 4.1)	3.6	(3.0, 4.3)	2.8	(1.7, 4.3)	
Saskatchewan	3.5	(2.9, 4.1)	3.5	(2.9, 4.2)	3.4	(2.2, 5.0)	
Alberta	14.5	(13.5, 15.7)	13.6	(12.4, 14.9)	18.9	(15.6, 22.7)	
British Columbia	11.1	(10.0, 12.2)	10.9	(9.7, 12.2)	12.1	(9.4, 15.3)	

The data source is the Canadian COVID-19 Antibody and Health Survey – Cycle 2. Estimates for Canada exclude the territories. All estimates are weighted and are percentages unless otherwise indicated

*CI* confidence interval, *COVID-19* coronavirus disease 2019, *n* unweighted sample, *PCC* post COVID-19 condition

^a^Suppressed for confidentiality or poor reliability

^b^Other ethnicity includes multiple ethnic origins, Inuit only, multiple Indigenous identities, unspecified Indigenous identity, and unknown ethnicity

**Table 2:** Health characteristics of adults self-reporting a positive COVID-19 test three or more months prior to questionnaire completion by post COVID-19 condition status, Canada, January 2020 to August 2022

Characteristics	All *n* = 4053		No PCC *n* = 3328		PCC *n* = 725		*p*-value
	Estimate	95% CI	Estimate	95% CI	Estimate	95% CI	
Current smoking status							0.0529
Non-smoker	90.6	(89.3, 91.8)	90.3	(88.8, 91.6)	92.1	(89.3, 94.4)	
Smokes less than daily	1.9	(1.4, 2.6)	1.7	(1.2, 2.4)	2.8	(1.3, 5.2)	
Smokes daily	7.5	(6.5, 8.6)	8.0	(6.8, 9.3)	5.1	(3.4, 7.3)	
Body mass index (kg/m^2^)^a^							0.0010
Underweight or normal (≤ 24.9)	31.1	(29.3, 33.0)	31.4	(29.4, 33.5)	29.7	(25.2, 34.6)	
Overweight (25.0 to 29.9)	36.1	(34.2, 38.1)	36.6	(34.5, 38.8)	33.7	(29.1, 38.5)	
Obesity class I (30.0 to 34.9)	18.4	(16.8, 20.0)	18.7	(16.9, 20.4)	17.0	(13.6, 20.8)	
Obesity class II and III (≥ 35.0)	11.9	(10.7, 13.2)	10.7	(9.4, 12.0)	17.8	(14.2, 21.7)	
Unknown	2.5	(1.9, 3.2)	2.6	(2.0, 3.4)	1.8	(0.9, 3.2)	
Cancer history							0.0072
Never diagnosed with cancer	91.5	(90.4, 92.5)	92.3	(91.2, 93.3)	87.9	(84.5, 90.7)	
Current cancer	1.1	(0.8, 1.6)	1.1	(0.7, 1.6)	1.2	(0.5, 2.3)	
Past cancer	3.9	(3.2, 4.7)	3.6	(2.9, 4.3)	5.5	(3.5, 8.2)	
Unknown current or past cancer diagnosis	3.4	(2.8, 4.2)	3.0	(2.3, 3.8)	5.5	(3.5, 8.0)	
Pre-existing chronic conditions							
Asthma	7.6	(6.6, 8.7)	6.2	(5.2, 7.4)	14.3	(11.0, 18.1)	< 0.0001
Chronic lung condition	1.5	(1.1, 2.0)	1.1	(0.7, 1.6)	3.6	(2.3, 5.4)	< 0.0001
Sleep apnea	5.1	(4.3, 5.9)	4.8	(4.0, 5.7)	6.4	(4.5, 8.7)	0.1198
High blood pressure	9.3	(8.4, 10.3)	8.4	(7.4, 9.5)	13.5	(10.6, 16.9)	0.0004
Diabetes	5.3	(4.5, 6.2)	5.1	(4.2, 6.2)	6.2	(4.3, 8.6)	0.3481
Chronic heart condition	1.7	(1.2, 2.3)	1.4	(1.0, 1.8)	3.3	(1.6, 6.0)	0.0088
Chronic kidney disease	0.6	(0.3, 1.1)	0.7	(0.3, 1.3)	-^b^	-	-
Liver disease	0.3	(0.2, 0.6)	0.4	(0.2, 0.7)	-	-	-
Chronic neurological disorder	1.5	(1.1, 2.0)	1.1	(0.7, 1.5)	3.6	(2.0, 6.1)	< 0.0001
Effects of a stroke	0.5	(0.3, 0.8)	0.5	(0.2, 0.8)	-	-	-
Alzheimer’s disease or other dementia	-	-	-	-	-	-	-
Chronic fatigue syndrome or fibromyalgia	1.3	(0.9, 1.8)	0.9	(0.6, 1.4)	3.1	(1.8, 5.1)	0.0001
Mental health condition	11.0	(9.8, 12.3)	9.9	(8.6, 11.4)	16.2	(12.8, 20.0)	0.0003
Osteoporosis	1.6	(1.2, 2.1)	1.3	(0.9, 1.8)	2.8	(1.5, 4.8)	0.0190
Arthritis	9.1	(8.1, 10.1)	8.2	(7.2, 9.3)	13.1	(10.3, 16.4)	0.0003
Back problems	9.3	(8.2, 10.5)	8.4	(7.2, 9.7)	13.6	(10.6, 17.1)	0.0004
Urinary incontinence	1.5	(1.1, 1.9)	1.1	(0.8, 1.6)	3.0	(1.7, 4.9)	0.0009
Bowel disorder	3.6	(2.9, 4.4)	2.9	(2.3, 3.7)	6.7	(4.2, 10.0)	0.0004
Chronic blood disorder	0.7	(0.4, 1.0)	0.5	(0.2, 0.9)	1.5	(0.8, 2.7)	0.0045
Weakened immune system	3.5	(2.8, 4.3)	2.5	(1.8, 3.3)	8.1	(5.6, 11.4)	< 0.0001
Other chronic condition	2.8	(2.2, 3.6)	2.1	(1.6, 2.7)	6.3	(3.9, 9.7)	< 0.0001
Number of pre-existing chronic conditions (mean)	0.8	(0.7, 0.8)	0.7	(0.6, 0.7)	1.3	(1.1, 1.4)	< 0.0001
Number of pre-existing chronic conditions							< 0.0001
0	58.2	(56.2, 60.2)	61.4	(59.2, 63.6)	42.7	(37.7, 47.8)	
1	23.4	(21.7, 25.2)	22.9	(21.0, 24.8)	25.8	(21.6, 30.3)	
2 to 3	13.9	(12.6, 15.3)	12.4	(11.0, 13.8)	21.4	(17.7, 25.4)	
4 +	4.5	(3.8, 5.3)	3.3	(2.7, 4.0)	10.2	(7.3, 13.6)	
Pre-existing chronic symptoms							
Pain	11.8	(10.5, 13.1)	10.1	(8.8, 11.4)	19.9	(15.9, 24.4)	< 0.0001
Headache	5.9	(5.0, 7.0)	4.5	(3.7, 5.5)	12.8	(9.5, 16.6)	< 0.0001
Shortness of breath	4.5	(3.7, 5.3)	3.4	(2.8, 4.2)	9.5	(6.7, 13.0)	< 0.0001
Cough	2.7	(2.1, 3.3)	2.3	(1.7, 3.0)	4.6	(3.1, 6.6)	0.0019
Difficulty speaking or hoarseness	0.5	(0.3, 0.8)	0.5	(0.3, 0.7)	0.9	(0.4, 1.9)	0.0620
Difficulty swallowing	0.7	(0.4, 1.1)	0.6	(0.3, 1.0)	-	-	-
Loss of appetite	0.8	(0.5, 1.1)	0.6	(0.4, 0.9)	1.8	(0.9, 3.1)	0.0014
Loss of taste or smell	0.7	(0.4, 1.0)	0.4	(0.2, 0.7)	2.1	(1.0, 3.7)	< 0.0001
Feeling thirsty	2.6	(2.0, 3.3)	2.2	(1.6, 2.9)	4.6	(3.1, 6.6)	0.0014
Nausea or vomiting	1.2	(0.7, 1.8)	0.8	(0.4, 1.3)	-	-	-
Upset stomach, bloating, gas	6.0	(5.1, 7.0)	5.2	(4.3, 6.2)	10.0	(7.1, 13.7)	0.0003
Heartburn or indigestion	6.4	(5.5, 7.4)	5.9	(5.0, 7.0)	8.5	(6.2, 11.3)	0.0276
Frequent urination	4.4	(3.7, 5.1)	3.7	(3.1, 4.5)	7.5	(5.5, 10.0)	< 0.0001
Irregular bowel movement	5.9	(5.0, 7.0)	5.2	(4.3, 6.3)	9.3	(6.6, 12.7)	0.0011
Change in body weight	2.8	(2.2, 3.5)	2.4	(1.8, 3.1)	4.9	(3.1, 7.3)	0.0026
Chest tightness	1.9	(1.3, 2.6)	1.4	(0.9, 2.0)	4.3	(2.1, 7.6)	0.0008
Symptoms relating to the heart (e.g., fast, pounding, or irregular heartbeat)	3.5	(2.8, 4.2)	2.4	(1.9, 3.1)	8.3	(5.8, 11.6)	< 0.0001
Fatigue, tiredness, loss of energy	12.4	(11.0, 13.8)	10.2	(8.8, 11.6)	23.0	(18.9, 27.6)	< 0.0001
General weakness	3.0	(2.3, 3.7)	2.3	(1.7, 3.0)	6.3	(4.1, 9.3)	< 0.0001
Feeling hot or cold	4.5	(3.7, 5.4)	3.2	(2.5, 4.0)	10.6	(7.6, 14.3)	< 0.0001
Numbness or tingling	4.7	(3.9, 5.6)	3.8	(3.1, 4.6)	9.1	(6.3, 12.7)	< 0.0001
Dizziness	3.5	(2.8, 4.4)	2.4	(1.8, 3.0)	9.3	(6.3, 13.0)	< 0.0001
Fainting	-	-	-	-	-	-	-
Swelling	2.2	(1.7, 2.9)	1.5	(1.1, 2.2)	5.4	(3.3, 8.3)	< 0.0001
Skin irritation	5.9	(4.9, 6.9)	5.1	(4.2, 6.2)	9.5	(6.6, 13.1)	0.0009
Joint inflammation	8.8	(7.7, 9.9)	7.4	(6.3, 8.6)	15.2	(11.8, 19.2)	< 0.0001
Stiffness	8.1	(7.1, 9.1)	6.4	(5.5, 7.5)	15.9	(12.4, 19.9)	< 0.0001
Difficulty falling or staying asleep	11.5	(10.3, 12.8)	9.4	(8.2, 10.7)	21.2	(17.2, 25.7)	< 0.0001
Difficulty thinking or problem solving (brain fog)	4.6	(3.7, 5.5)	3.7	(2.9, 4.6)	8.7	(6.0, 12.1)	< 0.0001
Confusion, memory loss	3.0	(2.4, 3.8)	2.2	(1.7, 2.9)	7.0	(4.6, 10.2)	< 0.0001
Loss of interest in activities	5.0	(4.2, 6.0)	3.7	(3.0, 4.5)	11.7	(8.5, 15.5)	< 0.0001
Sadness, pessimism, hopelessness, or depression	8.7	(7.5, 10.0)	7.3	(6.1, 8.6)	15.7	(12.1, 19.9)	< 0.0001
Stress or anxiety	20.2	(18.6, 21.9)	17.7	(16.0, 19.6)	32.3	(27.7, 37.1)	< 0.0001
Other chronic symptom	-	-	-	-	-	-	-
Number of pre-existing chronic symptoms (mean)	1.7	(1.6, 1.8)	1.4	(1.3, 1.5)	3.2	(2.6, 3.7)	< 0.0001
Number of pre-existing chronic symptoms							< 0.0001
0	54.7	(52.7, 56.8)	58.3	(56.0, 60.5)	37.7	(33.1, 42.5)	
1 to 2	23.4	(21.7, 25.1)	23.3	(21.5, 25.2)	23.9	(20.0, 28.2)	
3 to 5	13.0	(11.7, 14.3)	11.8	(10.5, 13.2)	18.6	(15.1, 22.4)	
6 +	8.9	(7.8, 10.1)	6.6	(5.6, 7.7)	19.8	(16.1, 24.1)	
Disability status							0.0014
Identifies as having a disability	5.6	(4.7, 6.5)	4.9	(4.0, 5.8)	9.1	(6.4, 12.3)	
Does not identify as having a disability	92.5	(91.4, 93.6)	93.5	(92.2, 94.6)	88.0	(84.4, 91.0)	
Unknown	1.9	(1.3, 2.7)	1.7	(1.0, 2.6)	3.0	(1.5, 5.3)	
Self-rated health							
Fair or poor health	6.9	(5.9, 7.9)	5.1	(4.2, 6.1)	15.4	(12.1, 19.2)	< 0.0001
Fair or poor mental health	10.4	(9.2, 11.8)	8.3	(7.0, 9.7)	20.7	(16.7, 25.3)	< 0.0001

The data source is the Canadian COVID-19 Antibody and Health Survey – Cycle 2. Estimates for Canada exclude the territories. All estimates are weighted and are percentages unless otherwise indicated

*CI* confidence interval, *COVID-19* coronavirus disease 2019, *n* unweighted sample, *PCC* post COVID-19 condition

^a^Body mass index, defined as a person’s weight in kilograms divided by the square of their height in metres, was corrected for known biases in self-reported height and weight (Shields et al., 2011) by Statistics Canada

^b^Suppressed for confidentiality or poor reliability

**Table 3:** COVID-19 vaccine and infection characteristics of adults self-reporting a positive COVID-19 test three or more months prior to questionnaire completion by post COVID-19 condition status, Canada, January 2020 to August 2022

Characteristics	All *n* = 4053		No PCC *n* = 3328		PCC *n* = 725		*p*-value
	Estimate	95% CI	Estimate	95% CI	Estimate	95% CI	
Number of vaccine doses as of questionnaire completion							0.3098
0	6.1	(5.0, 7.3)	6.4	(5.2, 7.7)	4.6	(2.6, 7.7)	
1	1.0	(0.5, 1.6)	0.9	(0.5, 1.7)	-^a^	-	
2	34.5	(32.6, 36.5)	33.8	(31.8, 36.0)	37.8	(33.0, 42.8)	
3 +	58.5	(56.4, 60.5)	58.9	(56.6, 61.1)	56.5	(51.5, 61.3)	
Number of vaccine doses received prior to the month of infection							< 0.0001
0	24.8	(22.9, 26.7)	21.7	(19.7, 23.7)	39.6	(34.8, 44.6)	
1	3.4	(2.7, 4.3)	3.4	(2.6, 4.4)	3.4	(1.9, 5.6)	
2	46.1	(44.1, 48.2)	47.9	(45.7, 50.1)	37.7	(33.1, 42.6)	
3	23.3	(21.7, 25.0)	24.5	(22.7, 26.4)	17.6	(14.0, 21.7)	
Unknown	2.4	(1.8, 3.1)	2.5	(1.9, 3.4)	1.6	(0.8, 2.8)	
Months since last vaccine dose prior to the month of infection							< 0.0001
No doses received prior to infection	24.7	(22.8, 26.7)	21.6	(19.7, 23.7)	39.6	(34.8, 44.6)	
1 to 3	25.2	(23.5, 26.9)	26.5	(24.6, 28.4)	18.7	(15.1, 22.8)	
4 to 6	32.9	(30.9, 34.8)	34.1	(31.9, 36.3)	27.1	(22.8, 31.7)	
7 +	14.3	(12.9, 15.7)	14.6	(13.1, 16.2)	13.0	(10.0, 16.5)	
Unknown	2.9	(2.3, 3.7)	3.2	(2.5, 4.1)	1.6	(0.8, 2.8)	
Number of vaccine doses received in the infection month to 3 months after							0.0030
0	68.6	(66.7, 70.4)	68.8	(66.7, 70.8)	67.8	(62.8, 72.5)	
1	25.7	(24.0, 27.5)	25.9	(24.0, 27.9)	24.6	(20.3, 29.4)	
2	3.2	(2.5, 4.0)	2.6	(1.9, 3.4)	5.9	(3.6, 8.9)	
Unknown	2.5	(2.0, 3.3)	2.7	(2.0, 3.5)	1.7	(0.9, 3.0)	
Number of vaccine doses received more than 3 months after the infection month							< 0.0001
0	76.8	(75.0, 78.6)	79.8	(77.9, 81.6)	62.8	(57.7, 67.7)	
1	7.1	(6.0, 8.2)	6.3	(5.2, 7.5)	11.0	(7.8, 15.0)	
2	8.3	(7.1, 9.6)	7.4	(6.2, 8.8)	12.3	(9.2, 16.1)	
3	5.9	(5.0, 6.9)	4.4	(3.6, 5.5)	12.7	(9.5, 16.6)	
Unknown	1.9	(1.4, 2.6)	2.1	(1.5, 2.9)	-	-	
Testing status							< 0.0001
Positive polymerase chain reaction	51.1	(49.1, 53.0)	48.6	(46.4, 50.7)	63.1	(58.3, 67.8)	
Positive rapid antigen test	48.9	(47.0, 50.9)	51.4	(49.3, 53.6)	36.9	(32.2, 41.7)	
Time period of infection^b^							< 0.0001
Jan 2020 to Dec 2020	10.7	(9.5, 12.2)	8.3	(7.1, 9.7)	22.4	(18.4, 26.9)	
Jan 2021 to Jun 2021	10.0	(8.7, 11.3)	9.0	(7.7, 10.4)	14.8	(11.1, 19.1)	
Jul 2021 to Nov 2021	5.6	(4.7, 6.6)	5.7	(4.6, 6.9)	5.3	(3.4, 7.7)	
Dec 2021 to May 2022	72.9	(70.9, 74.9)	76.2	(74.0, 78.3)	57.2	(52.1, 62.2)	
Unknown	0.7	(0.4, 1.2)	0.8	(0.4, 1.4)	-	-	
Hospitalized due to COVID-19							< 0.0001
No	96.4	(95.6, 97.1)	97.0	(96.1, 97.7)	93.7	(91.0, 95.7)	
Yes	2.3	(1.7, 3.0)	1.6	(1.0, 2.3)	5.6	(3.7, 8.2)	
Unknown	1.3	(0.9, 1.8)	1.5	(1.0, 2.0)	-	-	
Severity of initial infection symptoms							< 0.0001
No or mild symptoms—didn’t affect daily life	40.9	(38.9, 42.9)	46.6	(44.3, 48.8)	13.9	(10.9, 17.4)	
Moderate symptoms—some effect on daily life	42.6	(40.6, 44.6)	41.9	(39.6, 44.1)	46.2	(41.3, 51.1)	
Severe symptoms—significant effect on daily life or hospitalized	16.5	(14.9, 18.1)	11.6	(10.1, 13.2)	39.9	(35.2, 44.7)	

The data source is the Canadian COVID-19 Antibody and Health Survey – Cycle 2. Estimates for Canada exclude the territories. All estimates are weighted percentages

*CI* confidence interval, *COVID-19* coronavirus disease 2019, *n* unweighted sample, *PCC* post COVID-19 condition

^a^Suppressed for confidentiality or poor reliability

^b^Categorized to ascertain the impacts of emerging variants while acknowledging the limitations of sample size: Jan to Dec 2020 (wild type); Jan to Jun 2021 (alpha); Jul to Nov 2021 (delta); and Dec 2021 to May 2022 (omicron) (Government of Canada, 2022a). The last time period ended May 2022 instead of Aug 2022 to allow for 3 months of follow-up to ascertain PCC

For the outcomes severity and time to resolution of PCC, presence of each of 14 PCC symptoms, as well as the number of PCC symptoms, were also examined (Table 4). In addition, the limitations in daily activities variable was examined for time to resolution of PCC.

**Table 4:** Symptoms and limitations in daily activities reported by adults with post COVID-19 condition after a positive COVID-19 test, Canada, January 2020 to August 2022

Characteristics	Estimate	95% CI
Symptoms		
Shortness of breath or difficulty breathing	36.9	(32.1, 41.8)
Coughing	37.2	(32.4, 42.2)
Fever	7.6	(5.2, 10.7)
Headache	27.5	(23.1, 32.2)
Loss of taste or smell	30.2	(25.4, 35.2)
General weakness	29.1	(24.6, 34.0)
Fatigue, tiredness, or loss of energy	70.5	(65.7, 75.0)
Pain, excluding chest pain or headache (e.g., muscular, abdominal, joint)	21.6	(17.9, 25.8)
Chest pain	15.9	(12.4, 19.9)
Symptoms relating to the heart (e.g., fast, pounding, or irregular heartbeat)	13.2	(9.9, 17.1)
Difficulty thinking or problem solving (brain fog)	33.4	(28.7, 38.3)
Stress or anxiety	24.6	(20.4, 29.1)
Sadness, pessimism, hopelessness or depression	16.5	(13.0, 20.6)
Other symptoms	10.6	(8.1, 13.6)
Number of symptoms (mean)	3.7	(3.5, 4.0)
Number of symptoms		
1	22.9	(19.0, 27.1)
2 to 3	34.1	(29.5, 39.0)
4 to 6	26.7	(22.5, 31.2)
7 +	16.4	(12.8, 20.4)
Limited in daily activities^a^		
Never or rarely	47.3	(42.1, 52.4)
Sometimes	31.2	(26.8, 36.0)
Often or always	21.5	(17.6, 25.8)

The data source is the Canadian COVID-19 Antibody and Health Survey – Cycle 2. Estimates for Canada exclude the territories. All estimates are weighted and are percentages unless otherwise indicated

^a^Daily activities were defined as preparing meals, everyday housework, heavier household chores, getting to appointments and running errands, looking after personal finances, personal care and basic medical care at home, and moving around inside one’s residence

### Analyses

Estimates of the burden of PCC used confirmed and suspected infections to provide a range of plausible values. However, all other analyses were limited to confirmed infections. Statistical procedures that acknowledge the complex survey design were used in combination with final and bootstrap weights supplied by Statistics Canada. Descriptive statistics (mean, percentage) and corresponding 95% confidence intervals (CIs) were used to estimate the burden of PCC and characterize the population with confirmed infections overall and by PCC status. CIs for weighted means use the *t*-distribution, and the *t*-test was used to test for differences between group means (alpha = 0.05, two tailed). CIs for weighted proportions were calculated using the Clopper-Pearson (exact) method, and the design-based first-order Rao-Scott test of association was used to test for group differences.

To estimate the co-occurrence of PCC symptoms, the percentage of adults experiencing each of the 14 symptoms captured by the questionnaire was examined by the reported presence of each symptom and visualized using a heatmap constructed in Microsoft® Excel® for Microsoft 365.

Multivariable modeling employing complete case analysis was used to determine which factors were independently associated with the development (binomial logistic regression), severity (ordinal logistic regression), and resolution of PCC (Cox proportional hazards regression). Considering the large number of variables to be assessed combined with the increased sampling error associated with the analysis of complex survey data, a stepwise selection process was implemented (see supplementary methods). When the main effects models were established, interactions between each retained variable and sex were tested (alpha = 0.05, two-tailed), one at a time, using product terms. Interactions with sex were examined because of differences in the acute COVID-19 and PCC experiences of males and females, and previously identified interactions between sex, age, and the development of PCC (Jensen et al., 2022; Puntoni et al., 2023).

For the Cox proportional hazards modeling, the Efron approximation was used to handle the ties (Hertz-Picciotto & Rockhill, 1997), and the proportional hazards assumption for each retained variable was tested (alpha = 0.05, two-tailed) using time-dependent covariates. SAS Enterprise Guide 7.15 (SAS 9.4™) was used for all analyses. See supplementary methods for measures taken to ensure confidentiality and reliability of estimates.

### Ethics statement

Our study was exempt from research ethics board review under article 2.2 of the Tri-Council Policy Statement: Ethical Conduct for Research Involving Humans (Government of Canada, 2022b).

We used the STROBE reporting guideline for cross-sectional studies to ensure complete and adequate reporting of our research (von Elm et al., 2007).

## Results

### Cumulative incidence and prevalence of PCC

**Fig. 2:**
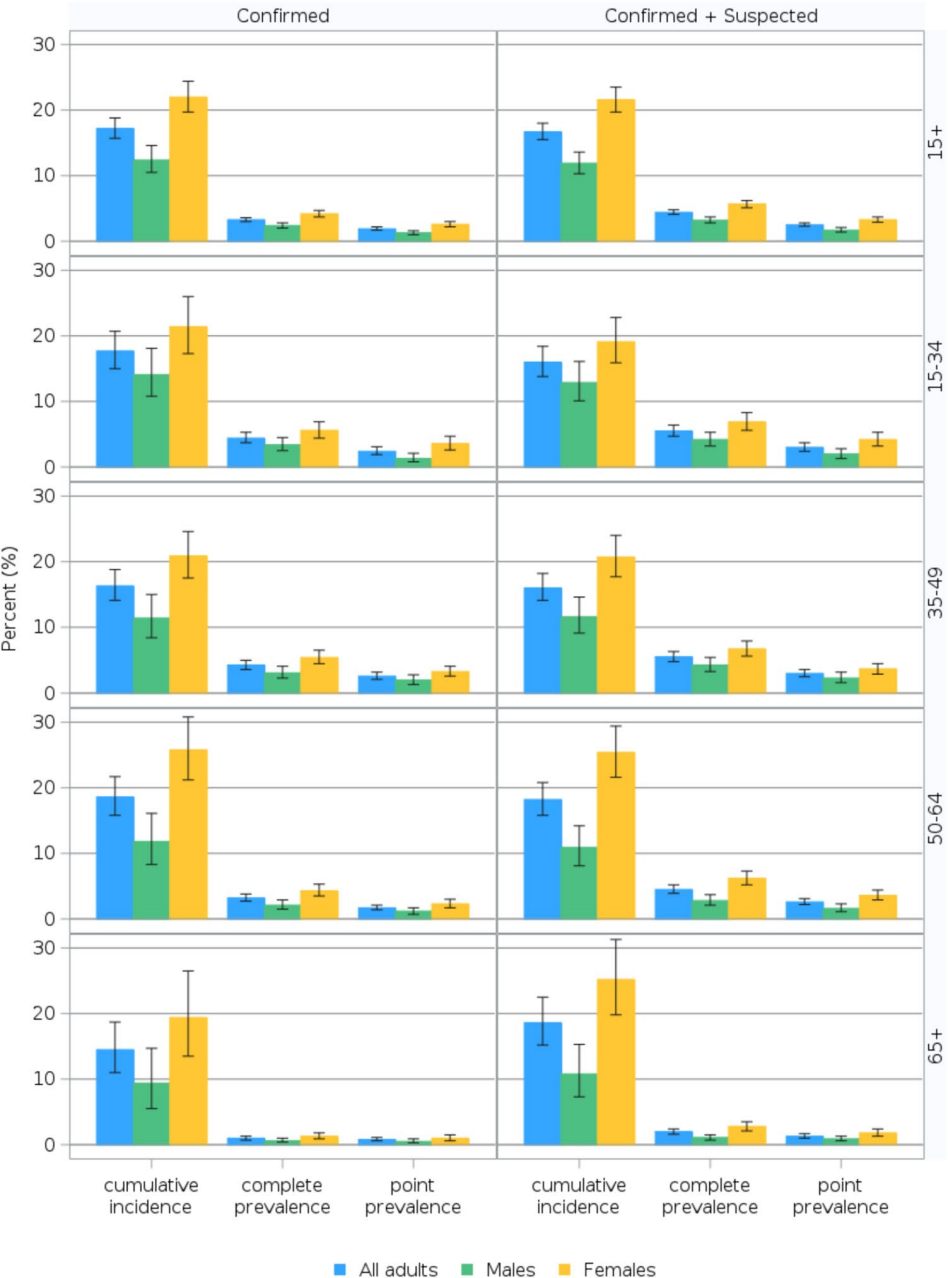
Incidence and prevalence of post COVID-19 condition among adults by COVID-19 test status, sex, and age groups, Canada, January 2020 to August 2022. The data source is the Canadian COVID-19 Antibody and Health Survey – Cycle 2. Estimates for Canada exclude the territories. All estimates are weighted percentages with 95% confidence intervals. For incidence, age refers to age at infection. For prevalence, age refers to age at questionnaire completion. All respondents were adults, aged 18 + years, at the time of questionnaire completion. *COVID-19* coronavirus disease 2019

As of August 2022, 29.9% (95% CI 29.1, 30.6) of adults in Canada had tested positive for COVID-19 and an additional 9.4% (95% CI 8.9, 9.9) suspected they had been infected for an overall combined infection rate of 39.3% (95% CI 38.4, 40.1). The method of calculating cumulative incidence did not substantially impact the overall estimates: 17.2% (95% CI 15.7, 18.8) of adults with confirmed infections and 16.7% (95% CI 15.5, 18.0) of adults with confirmed or suspected infections experienced PCC (Fig. 2, Table S1). This translates to 3.3% (95% CI 3.0, 3.6) and 4.4% (95% CI 4.1, 4.8) of all adults, respectively, experiencing PCC. The greatest absolute difference in cumulative incidence of PCC by method of calculation was noted for females aged 65 + years: 19.4% (95% CI 13.5, 26.5) for confirmed infections vs. 25.2% (95% CI 19.8, 31.3) for confirmed or suspected infections. Excepting point prevalence based on confirmed cases among 65 + -year-olds, females consistently reported a significantly higher burden of PCC compared to males irrespective of method of calculation and age group (*p* < 0.05).

### Factors associated with the development of PCC

Numerous sociodemographic (Table 1), health-related (Table 2), and vaccine- and infection-related factors (Table 3) were associated with PCC when examined in isolation. Of note, although the total number of COVID-19 vaccines received at the time of responding did not differ between those with and those without PCC, adults reporting PCC were more likely to be completely unvaccinated prior to infection (39.6% vs. 21.7%) (Table 3).

### Post COVID-19 condition symptoms and impact on daily activities

More than 30% of adults with PCC reported fatigue, tiredness, or loss of energy (70.5%), coughing (37.2%), shortness of breath or difficulty breathing (36.9%), difficulty thinking or problem solving (33.4%), and loss of taste or smell (30.2%) (Table 4). Overall, 77.1% experienced more than one symptom, with an average of 3.7 being reported, and 21.5% reported symptoms often or always limited their daily activities.

**Fig. 3:**
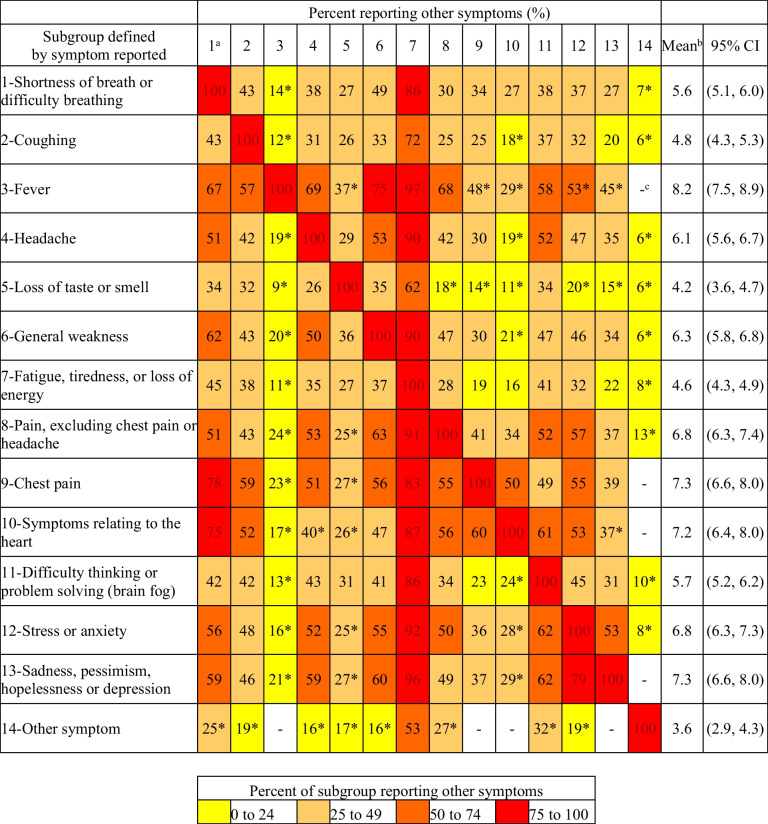
Heat map of symptoms reported by adults with post COVID-19 condition after a positive COVID-19 test by subgroups defined by symptom presence, Canada, January 2020 to August 2022. The data source is the Canadian COVID-19 Antibody and Health Survey – Cycle 2. Estimates for Canada exclude the territories. Estimates are percentages unless otherwise indicated. *CI* confidence interval; *COVID-19* coronavirus disease 2019. ^a^Numbers correspond to symptoms defined in the first column. ^b^Mean number of symptoms. ^c^Suppressed for confidentiality or poor reliability. *Interpret with caution: 16.6% < coefficient of variation ≤ 33.3%

Although the majority of adults with PCC reported more than one symptom, the presence of certain symptoms was associated with a higher average number of symptoms (Fig. 3). For example, adults reporting fever, chest pain, symptoms relating to the heart, or sadness, pessimism, hopelessness, or depression experienced, on average, seven or more symptoms. All four symptoms, however, were among the least commonly reported.

### Multivariable modeling

#### Characteristics associated with reporting PCC

Several demographic, health, and infection characteristics were independently significantly associated with reporting PCC after a positive COVID-19 test. The odds of PCC were 46% lower for males than for females (adjusted odds ratio (aOR) = 0.54, 95% CI 0.41, 0.70), 75% + higher for younger (< 65 years) than for older adults (65 + years), and 77% lower for Black than for white adults (aOR = 0.23, 95% CI 0.11, 0.51) (Table 5). The relative odds of PCC increased with the number of pre-existing chronic conditions from 1.75 (95% CI 1.26, 2.45) for one condition to 3.57 (95% CI 2.08, 6.13) for four or more. Pre-existing difficulty falling or staying asleep (aOR = 1.61, 95% CI 1.15, 2.25), loss of interest in activities (aOR = 2.46, 95% CI 1.53, 3.94), and loss of taste or smell (aOR = 6.15, 95% CI 2.38, 15.88) were all associated with increased odds of PCC. Adults infected after 2020 had 45–76% lower odds of PCC than those infected in 2020. The variable most strongly associated with PCC was severity of initial infection: relative to adults with no or mild symptoms, the odds for PCC were 3.52 (95% CI 2.58, 4.79) and 9.69 (95% CI 6.73, 13.96) times greater for adults with moderate and severe symptoms, respectively.

**Table 5:** Characteristics associated with development of post COVID-19 condition among adults with a positive COVID-19 test, Canada, January 2020 to August 2022 (*n* = 4043)

Characteristics	uOR	95% CI	aOR	95% CI	*p*-value^a^
Male sex (vs. female)	0.50	(0.40, 0.63)	0.54	(0.41, 0.70)	< 0.0001
Age at infection (years)					0.0129
15 to 34	1.27	(0.88, 1.83)	2.14	(1.34, 3.40)	
35 to 49	1.15	(0.81, 1.62)	1.93	(1.26, 2.97)	
50 to 64	1.35	(0.92, 1.96)	1.75	(1.12, 2.71)	
65 +	1.00		1.00		
Ethnicity					0.0008
White only	1.00		1.00		
First Nations only	2.64	(1.35, 5.16)	1.92	(0.91, 4.06)	
Metis only	0.75	(0.27, 2.06)	0.72	(0.21, 2.53)	
Black only	0.30	(0.15, 0.58)	0.23	(0.11, 0.51)	
Arab or West Asian only	0.54	(0.25, 1.16)	0.68	(0.27, 1.71)	
South Asian only	1.24	(0.75, 2.04)	1.19	(0.66, 2.13)	
East or Southeast Asian only	0.62	(0.37, 1.04)	0.71	(0.40, 1.27)	
Latin American only	0.65	(0.28, 1.50)	0.57	(0.23, 1.42)	
Other^b^	2.12	(1.10, 4.06)	2.79	(1.30, 5.99)	
Province of residence					0.0183
Newfoundland and Labrador	1.18	(0.73, 1.92)	1.75	(0.95, 3.20)	
Prince Edward Island	1.62	(1.04, 2.52)	2.05	(1.20, 3.51)	
Nova Scotia	1.18	(0.81, 1.71)	1.53	(0.96, 2.42)	
New Brunswick	1.05	(0.71, 1.56)	1.44	(0.89, 2.35)	
Quebec	0.85	(0.62, 1.15)	0.93	(0.65, 1.32)	
Ontario	1.00		1.00		
Manitoba	0.82	(0.55, 1.22)	0.84	(0.51, 1.38)	
Saskatchewan	1.02	(0.69, 1.51)	1.18	(0.75, 1.87)	
Alberta	1.45	(1.03, 2.02)	1.50	(1.02, 2.20)	
British Columbia	1.16	(0.79, 1.71)	1.29	(0.82, 2.04)	
Pre-existing chronic conditions					
Mental health condition	1.75	(1.29, 2.37)	0.61	(0.41, 0.91)	0.0144
Number of pre-existing chronic conditions					< 0.0001
0	1.00		1.00		
1	1.62	(1.23, 2.13)	1.75	(1.26, 2.45)	
2 to 3	2.48	(1.86, 3.31)	2.25	(1.55, 3.26)	
4 +	4.39	(2.88, 6.71)	3.57	(2.08, 6.13)	
Pre-existing chronic symptoms					
Difficulty falling or staying asleep	2.58	(1.93, 3.45)	1.61	(1.15, 2.25)	0.0060
Loss of interest in activities	3.48	(2.33, 5.20)	2.46	(1.53, 3.94)	0.0002
Loss of taste or smell	5.84	(2.39, 14.28)	6.15	(2.38, 15.88)	0.0002
Time period of infection^c^					< 0.0001
Jan to Dec 2020	1.00		1.00		
Jan to Jun 2021	0.61	(0.40, 0.95)	0.55	(0.32, 0.93)	
Jul to Nov 2021	0.34	(0.20, 0.59)	0.28	(0.15, 0.51)	
Dec 2021 to May 2022	0.28	(0.21, 0.38)	0.24	(0.17, 0.35)	
Unknown period of infection	-^d^	-	-	-	
Severity of initial infection symptoms					< 0.0001
No or mild symptoms—didn’t affect daily life	1.00		1.00		
Moderate symptoms—some effect on daily life	3.70	(2.75, 4.98)	3.52	(2.58, 4.79)	
Severe symptoms—significant effect on daily life or hospitalized	11.55	(8.35, 15.97)	9.69	(6.73, 13.96)	

The data source is the Canadian COVID-19 Antibody and Health Survey – Cycle 2. Estimates for Canada exclude the territories. All estimates are weighted. The reference category for a pre-existing chronic symptom or condition are adults without the chronic symptom or condition, respectively

*aOR* adjusted odds ratio, *CI* confidence interval, *n* unweighted number of respondents included in the final model, *uOR* unadjusted odds ratio

^a^*p*-value for the type 3 analysis of effect for a variable after adjusting for other variables retained in the final model

^b^Other ethnicity includes multiple ethnic origins, Inuit only, multiple Indigenous identities, unspecified Indigenous identity, and unknown ethnicity

^c^Categorized to ascertain the impacts of emerging variants while acknowledging the limitations of sample size: Jan to Dec 2020 (wild type); Jan to Jun 2021 (alpha); Jul to Nov 2021 (delta); and Dec 2021 to May 2022 (omicron) (Government of Canada, 2022a). The last time period ended May 2022 instead of Aug 2022 to allow for 3 months of follow-up to ascertain PCC

^d^Suppressed for confidentiality

#### Characteristics associated with greater limitations in daily activities

The odds of reporting greater limitations in daily activities were 46% lower for males than for females (aOR = 0.54, 95% CI 0.34, 0.85) and 2.31 times greater for those residing in a rural rather than an urban area (aOR = 2.31, 95% CI 1.35, 3.93) (Table 6). Adults reporting a disability (aOR = 2.87, 95% CI 1.14, 7.25), pre-existing chronic lung condition (aOR = 5.47, 95% CI 1.85, 16.12) or back problem (aOR = 2.34, 95% CI 1.26, 4.36), or experiencing PCC headache (aOR = 2.47, 95% CI 1.60, 3.83) or general weakness (aOR = 2.27, 95% CI 1.41, 3.68) had higher odds of greater limitations, while those experiencing PCC coughing had lower odds (aOR = 0.58, 95% CI 0.38, 0.88).

**Table 6:** Characteristics associated with greater limitations in daily activities among adults with post COVID-19 condition after a positive COVID-19 test, Canada, January 2020 to August 2022 (*n* = 718)

Characteristics	uOR	95% CI	aOR	95% CI	*p*-value^a^
Male sex (vs. female)	0.54	(0.36, 0.81)	0.54	(0.34, 0.85)	0.0084
Age at infection (years)					0.8910
15 to 34	0.56	(0.27, 1.17)	1.13	(0.52, 2.46)	
35 to 49	0.83	(0.42, 1.65)	1.29	(0.63, 2.65)	
50 to 64	0.84	(0.41, 1.71)	1.21	(0.57, 2.58)	
65 +	1.00		1.00		
Rural residence (vs. urban)	1.96	(1.23, 3.13)	2.31	(1.35, 3.93)	0.0021
Disability status					0.0151
Does not identify as having a disability	1.00		1.00		
Identifies as a person with a disability	3.03	(1.20, 7.65)	2.87	(1.14, 7.25)	
Unknown	-^b^	-	-	-	
Pre-existing chronic conditions					
Chronic lung condition	5.67	(2.72, 11.83)	5.47	(1.85, 16.12)	0.0021
Back problems	2.39	(1.34, 4.24)	2.34	(1.26, 4.36)	0.0071
Post COVID-19 condition symptoms					
Coughing	0.69	(0.48, 1.00)	0.58	(0.38, 0.88)	0.0108
Headache	2.90	(1.96, 4.30)	2.47	(1.60, 3.83)	< 0.0001
General weakness	2.76	(1.79, 4.27)	2.27	(1.41, 3.68)	0.0008

The data source is the Canadian COVID-19 Antibody and Health Survey – Cycle 2. Estimates for Canada exclude the territories. All estimates are weighted. Daily activities were defined as preparing meals, everyday housework, heavier household chores, getting to appointments and running errands, looking after personal finances, personal care and basic medical care at home, and moving around inside one’s residence. Limitations in daily activities was quantified as a trichotomy (never/rarely, sometimes, often/always) and modeled using cumulative logistic regression. The odds ratio indicates the effect of the variable on the odds of being in a more limited rather than less limited category. The reference category for conditions or symptoms are adults without the condition or symptom, respectively

*aOR* adjusted odds ratio, *CI* confidence interval, *COVID-19* coronavirus disease 2019, *n* unweighted number of respondents included in the final model, *uOR* unadjusted odds ratio

^a^*p*-value for the type 3 analysis of effect for a variable after adjusting for other variables retained in the final model

^b^Suppressed for confidentiality

#### Characteristics associated with recovery

Sex and age at infection significantly interacted in their relationship with recovery (Table 7). This interaction can be described in two complementary ways: age modified the relationship between sex and recovery; or sex modified the relationship between age and recovery. Using the first approach, among adults aged 15 to 34 years at infection, the rate of recovery was 2.26 (95% CI 1.09, 4.66) times greater among males than among females, but the aHR decreased with older age to 0.36 (95% CI 0.06, 2.06) among 65 + -year-olds. Using the second approach, younger age at infection (< 65 years) was associated with an increased rate of recovery among males, but not consistently so among females. Adults residing in households where the highest level of education completed was high school had a higher rate of recovery compared to those residing in households with a college or university education (aHR = 2.37, 95% CI 1.50, 3.74). Having two or more pre-existing chronic conditions relative to none or specific PCC symptoms (i.e., related to the heart, brain fog, or stress/anxiety) was associated with a greater than 50% reduction in the rate of recovery.

**Table 7:** Characteristics associated with recovery from post COVID-19 condition among adults with a positive COVID-19 test, Canada, January 2020 to August 2022 (*n* = 685)

Characteristics	uHR	95% CI	aHR	95% CI	*p*-value^a^
Male sex (vs. female)	1.32	(0.92, 1.89)	.	(.,.)	.
Age group at infection, years					.
15 to 34	2.92	(1.46, 5.86)	.	(.,.)	
35 to 49	2.68	(1.34, 5.37)	.	(.,.)	
50 to 64	3.45	(1.75, 6.80)	.	(.,.)	
65 +	1		1		
Highest education completed in household					0.0008
Less than high school	-^b^	-	-	-	
High school diploma	1.54	(0.95, 2.49)	2.37	(1.50, 3.74)	
Trade certificate or diploma	0.52	(0.23, 1.17)	0.5	(0.20, 1.29)	
College or university certificate, diploma, or degree	1		1		
Number of pre-existing chronic conditions					0.0005
0	1		1		
1	0.61	(0.41, 0.91)	0.77	(0.51, 1.16)	
2 to 3	0.34	(0.20, 0.57)	0.33	(0.18, 0.58)	
4 +	0.29	(0.12, 0.69)	0.38	(0.17, 0.86)	
Post COVID-19 condition symptoms					
Symptoms related to the heart (e.g., fast, pounding or irregular heartbeat)	0.14	(0.04, 0.47)	0.25	(0.07, 0.90)	0.0339
Difficulty thinking or problem solving (brain fog)	0.33	(0.19, 0.57)	0.44	(0.23, 0.86)	0.0167
Stress or anxiety	0.23	(0.13, 0.43)	0.48	(0.24, 0.96)	0.0387
Interaction between sex and age at infection					0.0492
Effect of sex by age at infection					
Male sex (vs. female) among 15- to 34-year-olds	.	(.,.)	2.26	(1.09, 4.66)	
Male sex (vs. female) among 35- to 49-year-olds	.	(.,.)	1.17	(0.62, 2.21)	
Male sex (vs. female) among 50- to 64-year-olds	.	(.,.)	0.69	(0.38, 1.24)	
Male sex (vs. female) among 65 + year-olds	.	(.,.)	0.36	(0.06, 2.06)	
Effect of age at infection by sex					
Age 15 to 34 vs 65 + years among males	.	(.,.)	9.61	(1.85, 49.93)	
Age 35 to 49 vs 65 + years among males	.	(.,.)	7.09	(1.32, 38.07)	
Age 50 to 64 vs 65 + years among males	.	(.,.)	6.34	(1.28, 31.36)	
Age 15 to 34 vs 65 + years among females	.	(.,.)	1.55	(0.58, 4.14)	
Age 35 to 49 vs 65 + years among females	.	(.,.)	2.2	(0.87, 5.53)	
Age 50 to 64 vs 65 + years among females	.	(.,.)	3.35	(1.38, 8.12)	

The data source is the Canadian COVID-19 Antibody and Health Survey – Cycle 2. Estimates for Canada exclude the territories. All estimates are weighted. Adjusted hazard ratios are not provided for effects involved in interactions and unadjusted hazard ratios are not provided for interactions

*aHR* adjusted hazard ratio, *CI* confidence interval, *n* unweighted number of respondents included in the final model, *uHR* unadjusted hazard ratio

^a^*p*-value for the type 3 analysis of effect for a variable after adjusting for other variables retained in the final model

^b^Suppressed for confidentiality

## Discussion

We found that, as of August 2022, about 16.7% of adults in Canada experienced PCC after a confirmed or suspected SARS-CoV-2 infection. Our estimate is lower than that based on the United States pulse survey (29.6%) as of September 2022 and higher than that based on the Australian COVID-19 Impact Monitoring Survey (9.7%) as of August 2022 (Biddle & Korda, 2022; National Center for Health Statistics, 2023), both of which included unconfirmed cases. As noted by Biddle and Korda, these differences may reflect the rise in COVID-19 cases relative to vaccination coverage: over the first 2 years of the pandemic, the cumulative confirmed COVID-19 cases per capita was highest in the United States followed by Canada and Australia.

Many of our findings regarding factors independently associated with the development of PCC in adults with confirmed infections are in line with a recent systematic review of adjusted associations. Tsampasian et al. (2023) found that female sex (OR = 1.56, 95% CI 1.41, 1.73), comorbidities, hospitalization (OR = 2.48, 95% CI 1.97, 3.13) or intensive care unit admission (OR = 2.37, 95% CI 2.18, 2.56), obesity (BMI ≥ 30 kg/m^2^, OR = 1.15, 95% CI 1.08, 1.23), and vaccination against COVID-19 with two doses (OR = 0.57; 95% CI 0.43, 0.76) were all associated with developing PCC. Jennings et al. (2023) also concluded that two or more doses of COVID-19 vaccine given pre-infection reduced the odds of developing PCC (OR = 0.67, 95% CI 0.60, 0.74). We found that obesity and vaccination status were significantly associated with PCC when examined in isolation, but were not retained in the final multivariable model, likely due to earlier selection of highly correlated variables. Unlike Tsampasian et al., who found that older age (≥ 40 years, OR = 1.21, 95% CI 1.11, 1.33) was associated with higher odds of PCC, we found that younger age was associated with higher odds of PCC among adults with confirmed infections (Table 5). Our finding of lower odds of PCC among adults infected later in the pandemic is consistent with a retrospective population-based cohort study of PCR confirmed cases conducted in Sweden that found higher rates of PCC for primary infections classified as wild type (HR = 6.31, 95% CI 5.64, 7.06), alpha (HR = 5.33, 95% CI 4.73, 5.99), and delta (HR = 3.26, 95% CI 2.80, 3.80) relative to omicron after adjusting for sociodemographics and comorbidities (Hedberg & Nauclér, 2024).

Studies examining factors associated with the resolution of PCC are limited. Early research on adults with confirmed COVID-19 indicated that male sex, younger age, less severe infection, infection with the omicron variant, and being fully vaccinated prior to initial infection may be associated with greater likelihood of remission, whereas the presence of “brain fog” or shortness of breath, history of cancer, history of tobacco consumption, higher body mass index, and greater number of symptoms during the acute phase may be associated with a slower resolution or lower likelihood of symptom resolution (Perlis et al., 2023; Robineau et al., 2022). Except for number of symptoms during the acute phase, we examined all of these factors, but common significant independent factors were limited to younger age, male sex, and brain fog (Table 7). Robineau et al. (2022) found that being older than 60 years reduced the rate of recovery by 22% (aHR = 0.78, 95% CI 0.68, 0.90) and being female reduced the rate of recovery by 36% (aHR = 0.64, 95% CI 0.58, 0.70), while we found sex and age interacted: the effect of younger age was stronger among males than among females and the effect of sex was statistically significant for the youngest age group only.

### Limitations

The primary strengths of this study are that it is population-based and considers a wide range of characteristics. Its primary limitations are its dependence on participation and self-report making it vulnerable to selection bias, recall error, inaccurate infection status information, question misinterpretation, and data capture errors (e.g., inputting, processing). Only 25.3% of adults invited to participate were included in the share file used for analysis. Although weights were adjusted for non-response and calibrated to reflect the target population using auxiliary information, the potential for biased estimates remains if those who participated and agreed to share their data systematically differed from the target population in ways not corrected through weighting. Due to limited testing capacity early in the pandemic, we estimated PCC burden using two approaches, one of which included suspected cases. Some suspected infections may have been the consequence of conditions or infections unrelated to SARS-CoV-2. Conversely, other respondents may have been unaware of a past SARS-CoV-2 infection and thus excluded from our analytic sample. Although respondents were asked about limitations in daily activities due to PCC, some may have found it difficult to disentangle the specific impact of PCC from other pre-existing chronic conditions and symptoms, resulting in limitations being inappropriately attributed to PCC. Similarly, some pre-existing chronic symptoms may have actually been PCC symptoms if respondents were unknowingly infected prior to their first reported infection, or if they incorrectly recalled or inputted the date the chronic symptom first started or the date of their first positive COVID-19 test. Consequently, some of the pre-existing chronic symptoms that were associated with the development of PCC may have actually been PCC symptoms. Finally, our CCAHS-2 share file did not include individual- or household-level income. Although area-based income and deprivation variables were available, sample design variables (e.g., clusters) and weights for each stage of selection were not, precluding an examination of these variables using multilevel modeling (Rabe-Hesketh & Skrondal, 2006; Zhu, 2014).

## Conclusion

Over the first two and a half years of the pandemic, a substantial proportion of adults with confirmed or suspected infections reported PCC, with females and people with comorbidities being disproportionately impacted. The best way to prevent PCC is to not get infected, by taking precautions appropriate for one’s risk level (e.g., masking). Initiatives to decrease the initial severity of infections, such as vaccinations and early post-infection anti-viral treatments, may prove useful in preventing or minimizing the impact of PCC. Further, certain PCC symptoms may help identify adults at greater risk of morbidity. We found that headache or general weakness were associated with greater limitations in daily activities, while symptoms relating to the heart, brain fog, and stress or anxiety were associated with a reduced rate of recovery. Although the risk of PCC after infection appeared to decrease over the time period examined, partly reflecting growing immunity, emergence of less virulent variants, and expanded testing, continued surveillance is needed because of the potential for novel immune-evading variants and the uncertain effect of multiple infections.

## Contributions to knowledge

What does this study add to existing knowledge?

- This is the first population-based study to comprehensively characterize PCC in Canada.
- Over the first two and a half years of the pandemic, an estimated 17.2% of adults reported PCC after a confirmed infection. Those afflicted experienced an average of 3.7 symptoms and 21.5% reported their symptoms often or always limited daily activities.
- Multivariable modeling indicated that females and people with comorbidities were disproportionately impacted. In addition, having a more severe initial infection increased the odds of PCC, and certain PCC symptoms were associated with greater limitations in daily activities and a reduced rate of recovery.

What are the key implications for public health interventions, practice, or policy?

- Initiatives to decrease the initial severity of infections, such as vaccinations and early post-infection anti-viral treatments, may prove useful in minimizing the burden of PCC.
- Certain PCC symptoms may help identify adults at greater risk of morbidity: headache or general weakness were associated with greater limitations in daily activities, while heart-related symptoms, brain fog, and stress or anxiety were associated with a decreased rate of recovery.
- Although the risk of PCC after infection appeared to decrease over the time period examined, continued surveillance is needed because of the potential for novel immune-evading variants and the uncertain effect of multiple SARS-CoV-2 infections.

## Supplementary Information

Below is the link to the electronic supplementary material.

Supplementary file1 (DOCX 21 KB)

## Data Availability

Canadian COVID-19 Antibody and Health Survey—Cycle 2 data are available through Statistics Canada’s Research Data Centres (https://www.statcan.gc.ca/eng/microdata/data-centres).

## Code availability

All analyses were completed using SAS Enterprise Guide 7.1 (SAS 9.4™).

## Declarations

### Disclaimer

The analyses, conclusions, opinions, and statements expressed herein are solely those of the authors. No endorsement by the Public Health Agency of Canada is intended or inferred.
