# Supplementary material for: Factors associated with the development, severity, and resolution of post COVID-19 condition in adults living in Canada, January 2020 to August 2022
Source: Can J Public Health. 2024 Nov 1;116(2):290–308. doi: 10.17269/s41997-024-00958-7 (PMC12075033; doi:10.17269/s41997-024-00958-7)
Supplement: Supplementary file 1 — Supplementary file1 (DOCX 21 KB) [file 41997_2024_958_MOESM1_ESM.docx]

**Supplementary Methods**

*Imputations*

*Diagnosed chronic conditions and symptoms*

Adults not completing the chronic condition section of the questionnaire were assumed to have none of the conditions. When the date of diagnosis of a chronic condition was completely missing, the chronic condition was assumed to have been diagnosed prior to the date of SARS-CoV-2 infection. A similar approach was used for chronic symptoms. These assumptions affected 4.0% and 4.1% of the respondents used in this study, respectively.

*Month and Year of COVID-19 vaccine doses*

Approximately 3.4% of all survey respondents reported receiving vaccine doses prior to the first vaccine dose administered in Canada on December 14, 2020. This percentage is too high to be explained by participation in vaccination trials. Examination of the data suggested participants may have misreported the year as 2020 rather than 2021. To correct for this issue, when the first two or three vaccine dose dates were complete and occurred in 2020 and the first vaccine dose was received prior to December 2020, all vaccine doses received in 2020 were shifted forward by one year. For all other respondents, if the first vaccine dose was received prior to December 2020 or in 2020 without a specified month, the first vaccine date was set to December 2020. Thereafter, dates were imputed, as necessary, to maintain the appropriate sequencing of vaccines: the second vaccine dose was assumed to occur two months after the first and the third vaccine dose was assumed to occur six months after the second. These intervals were the most common and median intervals between dose one and two and dose two and three, respectively. In total, 3.1% of respondents used in this study had 1 or more vaccine dose dates imputed.

*Multivariable modeling*

Sex at birth and age group at infection were always retained in the model. All other variables associated with the outcome of interest at an alpha level of 0.10 (two-tailed) during univariable modeling were added one at a time based on the rankings of univariable p-values. If the added variable was significant at an alpha level of 0.05 (two-tailed) after adjusting for previously selected variables, it was retained, otherwise it was excluded from further consideration. If the addition of a variable resulted in a previously selected variable becoming non-significant (p>0.05), the non-significant variable was permanently removed from the model. This process continued until all initially eligible variables were assessed.

*Confidentiality and reliability of estimates*

To ensure confidentiality, all descriptive estimates based on fewer than 30 respondents or having fewer than five respondents in the numerator of a proportion (unweighted) are suppressed. To ensure adequate reliability, descriptive estimates are suppressed if the coefficient of variation is greater than 33.3%. When 95% CIs are not presented, estimates with a coefficient of variation greater than 16.6% are flagged. For multivariable modeling results, variable categories with fewer than 30 respondents or five events (unweighted) are also suppressed.

| **Table S1** Incidence and prevalence of post COVID-19 condition among adults by COVID-19 test status, sex and age group, Canada, January 2020 to August 2022 | | | | | | |
| --- | --- | --- | --- | --- | --- | --- |
|  | Tested positive for COVID-19  n=4053 | | | Tested positive for COVID-19  or suspected an infection  n=5558 | | |
| Characteristic | Cumulative incidence | Complete prevalence | Point prevalence | Cumulative Incidence | Complete prevalence | Point prevalence |
| All adults | 17.2^a^  (15.7, 18.8) | 3.3  (3.0, 3.6) | 1.9  (1.7, 2.2) | 16.7  (15.5, 18.0) | 4.4  (4.1, 4.8) | 2.5  (2.3, 2.8) |
| Age (years)^b^ |  |  |  |  |  |  |
| 15-34 | 17.7  (15.0, 20.7) | 4.4  (3.7, 5.3) | 2.4  (1.9, 3.1) | 16.0  (13.8, 18.4) | 5.5  (4.7, 6.4) | 3.0  (2.4, 3.7) |
| 35-49 | 16.3  (14.1, 18.8) | 4.3  (3.6, 5.0) | 2.6  (2.1, 3.2) | 16.0  (14.1, 18.2) | 5.5  (4.8, 6.3) | 3.0  (2.5, 3.6) |
| 50-64 | 18.6  (15.8, 21.7) | 3.2  (2.7, 3.8) | 1.7  (1.4, 2.1) | 18.2  (15.8, 20.8) | 4.5  (3.9, 5.2) | 2.6  (2.2, 3.1) |
| 65+ | 14.5  (11.0, 18.7) | 1.0  (0.7, 1.3) | 0.8  (0.6, 1.1) | 18.6  (15.2, 22.5) | 2.0  (1.6, 2.4) | 1.3  (1.0, 1.7) |
|  |  |  |  |  |  |  |
| Males | 12.4  (10.5, 14.6) | 2.4  (2.0, 2.8) | 1.3  (1.0, 1.6) | 11.9  (10.3, 13.6) | 3.2  (2.8, 3.7) | 1.7  (1.4, 2.1) |
| Age (years) |  |  |  |  |  |  |
| 15-34 | 14.1  (10.8, 18.1) | 3.4  (2.5, 4.5) | 1.3  (0.8, 2.1) | 12.9  (10.1, 16.1) | 4.2  (3.2, 5.3) | 2.0  (1.3, 2.8) |
| 35-49 | 11.4  (8.4, 15.0) | 3.1  (2.3, 4.1) | 2.0  (1.3, 2.8) | 11.6  (9.1, 14.6) | 4.3  (3.3, 5.4) | 2.3  (1.6, 3.2) |
| 50-64 | 11.8  (8.3, 16.1) | 2.1  (1.5, 2.9) | 1.2  (0.7, 1.7) | 10.9  (8.1, 14.2) | 2.8  (2.1, 3.7) | 1.6  (1.1, 2.3) |
| 65+ | 9.4  (5.5, 14.7) | 0.6  (0.4, 1.0) | 0.5  (0.3, 0.9) | 10.8  (7.3, 15.3) | 1.1  (0.7, 1.5) | 0.9  (0.6, 1.3) |
|  |  |  |  |  |  |  |
| Females | 22.0  (19.7, 24.4) | 4.2  (3.7, 4.7) | 2.6  (2.2, 3.0) | 21.6  (19.7, 23.5) | 5.7  (5.1, 6.2) | 3.3  (2.9, 3.7) |
| Age (years) |  |  |  |  |  |  |
| 15-34 | 21.4  (17.3, 26.0) | 5.6  (4.4, 6.9) | 3.6  (2.6, 4.7) | 19.1  (15.9, 22.8) | 6.9  (5.6, 8.3) | 4.2  (3.2, 5.3) |
| 35-49 | 20.9  (17.5, 24.6) | 5.4  (4.5, 6.5) | 3.3  (2.6, 4.1) | 20.7  (17.7, 24.0) | 6.7  (5.6, 7.9) | 3.7  (2.9, 4.5) |
| 50-64 | 25.8  (21.2, 30.8) | 4.3  (3.5, 5.3) | 2.3  (1.7, 3.0) | 25.4  (21.6, 29.4) | 6.2  (5.2, 7.3) | 3.6  (2.9, 4.4) |
| 65+ | 19.4  (13.5, 26.5) | 1.3  (0.9, 1.8) | 1.0  (0.6, 1.5) | 25.2  (19.8, 31.3) | 2.8  (2.1, 3.5) | 1.8  (1.3, 2.4) |
| Note: The data source is the Canadian COVID-19 Antibody and Health Survey – Cycle 2. Estimates for Canada exclude the territories. COVID-19 = coronavirus disease 2019, n = unweighted sample.  ^a^All estimates are weighted percentages with 95% confidence intervals.  ^b^For incidence, age refers to age at infection. For prevalence, age refers to age at questionnaire completion. All respondents were adults (aged 18+) at the time of questionnaire completion. | | | | | | |
